# Risk factors for development of nonalcoholic fatty liver disease after pancreatoduodenectomy

**DOI:** 10.1002/ags3.12024

**Published:** 2017-07-20

**Authors:** Yoshiro Fujii, Atsushi Nanashima, Masahide Hiyoshi, Naoya Imamura, Koichi Yano, Takeomi Hamada

**Affiliations:** ^1^ Department of Hepato‐Biliary‐Pancreatic Surgery Faculty of Medicine University of Miyazaki Miyazaki Japan

**Keywords:** nonalcoholic fatty liver disease, pancreatoduodenectomy, risk factor

## Abstract

Considerable attention has been focused on nonalcoholic fatty liver disease (NAFLD) which occasionally develops after pancreatoduodenectomy (PD). The present study aimed to clarify the prevalence, sequential change in properties and risk factors for NAFLD development after PD. We enrolled 196 patients who underwent PD and a computed tomography (CT) scan 1 month, 6 months and 1 year after surgery. NAFLD was defined as a liver‐to‐spleen attenuation ratio on plain CT of <0.9. We compared various clinical factors between the NAFLD group and the control group. Individual prevalence of NAFLD at 1 month, 6 months and 1 year after surgery was 12%, 21% and 15%. Significantly different factors by univariate analysis were as follows: 1 month: age, sex, total protein (TP), total cholesterol (TC) and copper (Cu); 6 months: sex, disease, surgical method, portal vein resection (PVR), frequency of defecation, TC and Cu; 1 year: age, sex, disease, surgical method, PVR, frequency of defecation, TP and Cu. Risk factors by multivariate analysis were as follows: 1 month: not elderly age, female sex and a decrease in Cu; 6 months: female sex and a decrease in Cu; 1 year: a decrease in Cu. NAFLD after PD frequently developed in women with a decrease in serum Cu and was influenced by various factors related to poor digestion and absorption associated with pancreatic exocrine insufficiency.

## 1 INTRODUCTION

Pancreatoduodenectomy (PD) is widely accepted as a standard procedure for the treatment of periampullary diseases. Recent advances in surgical technique and perioperative management have reduced the morbidity and mortality of PD. Moreover, the establishment of adjuvant chemotherapy for pancreatic cancer has contributed to improved survival rate after surgery,^1^ and more patients have been able to survive for several years longer than ever before. Therefore, long‐term metabolic complications have drawn considerable attention, and clinical studies have reported that hepatic steatosis occasionally occurs in patients who undergo PD.^2^, ^3^, ^4^, ^5^, ^6^, ^7^ This steatosis received little interest before the last decade because the patients did not usually have severe hepatic dysfunction clinically even though a few reports documented some cases of hepatic failure after pancreatic resection.^8^, ^9^, ^10^ The prevalence of this steatosis was reported to range from 7.8% to 37%,^2^, ^3^, ^4^, ^5^, ^6^, ^11^, ^12^ but a few reports are available on the time of occurrence and sequential change in the properties of this complication.^13^ Etiology of this steatosis may be different from that of conventional nonalcoholic fatty liver disease (NAFLD) related to obesity, dyslipidemia or diabetes mellitus (DM). Post‐PD NAFLD is believed to be most commonly a result of insufficiency of digestion, absorption, nutrition and pancreatic exocrine function, but clarification of this etiology remains an important unsolved problem. Moreover, risk factors for NAFLD at specific points in time after PD have yet to be identified, so elucidation of these factors will contribute to the prevention and management of this complication. To investigate the prevalence and risk factors for NAFLD after PD, we retrospectively studied patients undergoing PD in our hospital.

## 2 METHODS

### 2.1 Patient selection

From June 2003 through December 2015, 301 patients underwent PD for periampullary disease in the Department of Hepato‐Biliary‐Pancreatic Surgery, Miyazaki University Hospital. Of these patients, 203 received a computed tomography (CT) scan until 1 year after surgery, and the remainder who either did not receive regular abdominal CT follow up or died within 1 year of PD were excluded from this study. Seven patients with hepatitis B virus surface antigen or antihepatitis C virus antibody were also excluded. Thus, 196 patients were enrolled in the present study, and they underwent physical, radiological and blood examinations at 1 month, 6 months and 1 year after PD. No patient had regular alcohol intake over 140 g/week after surgery, who was excluded from this study. Informed consent for this retrospective analysis was obtained by the opt‐out method, and this study was approved by the institutional review board at Miyazaki University in 2016 (approval number #2016‐081).

### 2.2 Background of patients

Patients had a diagnosis of pancreatic ductal cancer (PDC, n*=*70), distal bile duct cancer (n*=*46), ampullary neoplasm (n*=*35), intraductal papillary mucinous neoplasm of the pancreas (n*=*23), duodenal cancer (n*=*6), gallbladder cancer (n*=*3), chronic or autoimmune pancreatitis (n*=*6) and other miscellaneous diseases (n*=*7). To analyze the differences in the rate of NAFLD development according to disease type, we summarized two types of disease: PDC in 70 and other periampullary diseases in 126. Surgical procedures included pylorus‐preserving pancreatoduodenectomy (PPPD) in 151, subtotal stomach‐preserving pancreatoduodenectomy (SSPPD) in 30 and conventional pancreatoduodenectomy (Whipple's PD) in 15. Nine patients underwent combined surgery (hepatectomy in 5, colectomy in 3 and total gastrectomy in 1), and 17 underwent portal vein resection (PVR) with reconstruction.

### 2.3 Definition of NAFLD

**Figure 1:**
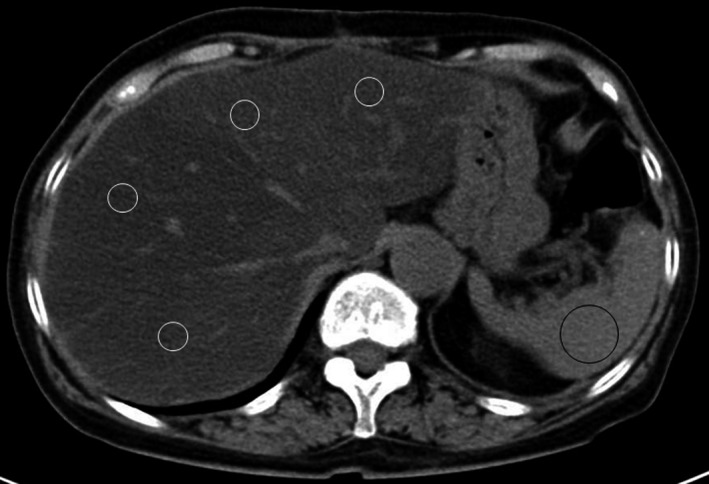
We measured the average computed tomography (CT) attenuation values in four sectors of the liver and the median value from all sections of the spleen on unenhanced CT scans. Nonalcoholic fatty liver disease was defined as a liver‐to‐spleen attenuation ratio of <0.9 or a hepatic attenuation value at least 10 Hounsfield units lower than the spleen attenuation value.

CT images were obtained with a 128‐row multidetector CT scanner (Siemens, Munich, Germany) using 120 kVp, 0.7 pitch, 5 mm slice thickness and 0.5 s scan time. NAFLD was defined as a liver‐to‐spleen attenuation ratio of <0.9^2^, ^3^, ^14^ or a hepatic attenuation value at least 10 Hounsfield units lower than the spleen attenuation value^2^, ^15^ on unenhanced abdominal CT scan. We used average CT attenuation values in four sectors of the liver and the median value from all sections of the spleen (Figure 1). Prevalence of NAFLD was evaluated at 1 month, 6 months and 1 year after PD.

### 2.4 Pancreatic enzymes

Pancreatic enzyme supplements such as Toughmac^®^ 3 g/day (Ono Pharmaceutical Co, Ltd, Osaka, Japan) or Berizym^®^ 3 g/day (Shionogi & Co. Ltd, Osaka, Japan) were routinely given in 124 patients. Since 2012, we have used the pancrelipase delayed‐release supplement LipaCreon^®^ 1200 mg/day (Eisai Co. Ltd, Tokyo, Japan) in 59 patients. At the attending physician's discretion, the remaining 13 patients did not receive any type of pancreatic enzyme supplement. Thus, to analyze the difference in the rate of NAFLD development according to the types of drug supplement given, we classified the patients into the following three groups: none (n*=*13), conventional drugs (n*=*124) and pancrelipase (n*=*59).

### 2.5 Clinical parameters

To investigate the clinical parameters associated with an occurrence of NAFLD after PD, we compared the following data between the patients with and without NAFLD at three time points: preoperatively: age, sex, body mass index (BMI), type of disease (PDC/other diseases), DM and dyslipidemia; perioperatively: surgical method (PPPD/SSPPD/PD), combined surgery, PVR and frequency of defecation at discharge from initial hospital stay; and postoperatively: types of pancreatic enzyme supplement and blood tests. Blood tests included serum values of total protein (TP, g/dL), total cholesterol (TC, mg/dL), homeostasis model assessment for insulin resistance (HOMA‐R), pancreatic function diagnostic test value (%), zinc (Zn, μg/dL) and copper (Cu, μg/dL). HOMA‐R value was calculated as fasting glucose (mg/dL) × immunoreactive insulin (μU/mL)/405. Pancreatic function diagnostic test was analyzed by the ratio of the 6‐hour urinary chemical recovery of para‐aminobenzoic acid.

### 2.6 Statistical analysis

Clinical parameters are expressed as the number (percentage) or median (range). Comparisons between groups were made using the χ^2^ test for categorical variables and the Mann‐Whitney *U*‐test for continuous variables. Multivariate logistic regression analysis by backward elimination method was also conducted to determine the risk factors associated with the development of NAFLD after PD. All *P* values were based on a two‐sided test, and a *P* value <.05 was considered to be statistically significant. Statistical analyses were carried out using SPSS software version 18.0 for Windows (SPSS, Chicago, IL, USA).

## 3 RESULTS

Nonalcoholic fatty liver disease developed before surgery in one patient only. This patient had advanced pancreatic cancer invading the duodenum, which caused duodenal obstruction and severe atrophy of the distal pancreas. NAFLD in this patient was improved at 1 month after PD and did not recur from 6 months after PD.

**Figure 2:**
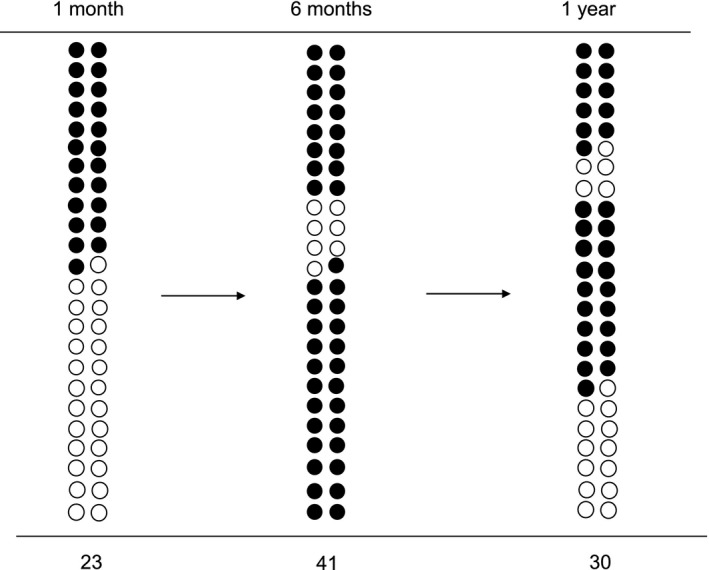
Patients with or without nonalcoholic fatty liver disease (NAFLD) were plotted as filled or open circles, respectively. NAFLD development was improved sequentially from 1 month to 6 months or 1 year after surgery in several patients. In contrast, NAFLD developed 6 months after surgery in others and was improved at 1 year after pancreatoduodenectomy in some patients.

Development of NAFLD at 1 month, 6 months and 1 year after PD was detected in 23 (12%), 41 (21%) and 30 (15%) patients, respectively. Of the 41 patients in whom NAFLD developed 6 months after PD, 18 recovered spontaneously by 1 year after, and 25 had not suffered from NAFLD 1 month after PD. Sequential change in patients with NAFLD at 1 month, 6 months and 1 year after PD is summarized in Figure 2.

Comparison of laboratory data between the patients with and without NAFLD by univariate analysis showed a significant decrease in age, BMI, levels of serum TP, TC and Cu 1 month after PD in the NAFLD group. Additionally, by univariate analysis, sex and rate of DM were significantly different between the two groups (Table 1).

**Table 1:** Risk factors for NAFLD 1 month after PD by univariate analysis

	NAFLD (n=23)	Control (n=173)	*P* value
Age (years)	63±11	68±9	.010
Sex (male/female)	4/19	126/47	<.001
BMI (kg/m^2^)	20±3	22±3	.013
Disease (PDC/Other diseases)	12/11	58/115	.080
DM (%)	0	20	.019
Dyslipidemia (%)	0	13	.070
Surgical method (PPPD/SSPPD/PD)	17/5/1	134/25/14	.574
Combined surgery (%)	13	3	.039
Portal vein resection (%)	9	9	.997
Frequency of defecation (/day)	1.8±1.2	1.4±1.0	.148
Pancreatic enzymes	1/15/7	12/109/52	.895
(None/Conventional drugs/Pancrelipase)			
Total protein (g/dL)	5.9±0.6	6.2±0.6	.014
Total cholesterol (mg/dL)	113±32	128±30	.021
PFD (%)	45±15	43±19	.574
HOMA‐R	0.64±0.4	0.92±0.6	.052
Zinc (μg/dL)	102±22	93±17	.097
Copper (μg/dL)	82±20	108±21	<.001

BMI, body mass index; DM, diabetes mellitus; HOMA‐R, homeostasis model assessment for insulin resistance; NAFLD, nonalcoholic fatty liver disease; PD, pancreatoduodenectomy; PDC, pancreatic ductal cancer; PFD, pancreatic function diagnostic test; PPPD, pylorus‐preserving pancreatoduodenectomy; SSPPD, subtotal stomach‐preserving pancreatoduodenectomy.

At 6 months after PD, sex, type of disease, surgical method and PVR rate were significantly different between the two groups by univariate analysis. Frequency of defecation was increased and levels of serum TC and Cu were decreased significantly in the NAFLD group by univariate analysis (Table 2).

**Table 2:** Risk factors for NAFLD 6 months after PD by univariate analysis

	NAFLD (n=41)	Control (n=155)	*P* value
Age (years)	65±11	68±9	.081
Sex (male/female)	19/22	111/44	.002
BMI (kg/m^2^)	22±3	22±3	.310
Disease (PDC/Other diseases)	25/16	45/110	<.001
DM (%)	20	17	.681
Dyslipidemia (%)	10	12	.738
Surgical method (PPPD/SSPPD/PD)	25/12/4	126/18/11	.013
Combined surgery (%)	10	3	.076
Portal vein resection (%)	20	6	.006
Frequency of defecation (/day)	2.0±1.7	1.4±0.7	.028
Pancreatic enzymes	3/27/11	10/97/48	.871
(None/Conventional drugs/Pancrelipase)			
Total protein (g/dL)	6.4±0.7	6.5±0.6	.312
Total cholesterol (mg/dL)	128±30	147±29	<.001
PFD (%)	45±17	47±16	.493
HOMA‐R	1.37±1.4	1.00±0.7	.197
Zinc (μg/dL)	62±10	65±13	.362
Copper (μg/dL)	77±25	93±22	.009

BMI, body mass index; DM, diabetes mellitus; HOMA‐R, homeostasis model assessment for insulin resistance; NAFLD, nonalcoholic fatty liver disease; PD, pancreatoduodenectomy; PDC, pancreatic ductal cancer; PFD, pancreatic function diagnostic test; PPPD, pylorus‐preserving pancreatoduodenectomy; SSPPD, subtotal stomach‐preserving pancreatoduodenectomy.

At 1 year after PD, age and levels of serum TP and Cu were decreased, and frequency of defecation was increased significantly in the NAFLD group by univariate analysis. Sex, type of disease, surgical method and PVR rate were also significantly different between the two groups by univariate analysis (Table 3).

**Table 3:** Risk factors for NAFLD 1 year after PD by univariate analysis

	NAFLD (n=30)	Control (n=166)	*P* value
Age (years)	62±10	69±9	<.001
Sex (male/female)	10/20	120/46	<.001
BMI (kg/m^2^)	22±3	22±3	.848
Disease (PDC/Other diseases)	20/10	50/116	<.001
DM (%)	20	17	.677
Dyslipidemia (%)	10	11	.817
Surgical method (PPPD/SSPPD/PD)	20/9/1	131/21/14	.042
Combined surgery (%)	3	5	.720
Portal vein resection (%)	27	5	<.001
Frequency of defecation (/day)	2.3±1.9	1.3±0.7	.013
Pancreatic enzymes	1/20/9	12/104/50	.724
(None/Conventional drugs/Pancrelipase)			
Total protein (g/dL)	6.4±0.7	6.6±0.6	.025
Total cholesterol (mg/dL)	139±29	149±33	.137
PFD (%)	44±16	49±22	.306
HOMA‐R	1.06±0.9	0.98±0.6	.652
Zinc (μg/dL)	69±19	70±13	.738
Copper (μg/dL)	73±31	98±23	.001

BMI, body mass index; DM, diabetes mellitus; HOMA‐R, homeostasis model assessment for insulin resistance; NAFLD, nonalcoholic fatty liver disease; PD, pancreatoduodenectomy; PDC, pancreatic ductal cancer; PFD, pancreatic function diagnostic test; PPPD, pylorus‐preserving pancreatoduodenectomy; SSPPD, subtotal stomach‐preserving pancreatoduodenectomy.

Age (not elderly), female sex and a decrease in Cu were related to an increased risk of developing NAFLD 1 month after PD by multivariate analysis (Table 4). Multivariate analysis also showed female sex and a decrease in serum Cu 6 months after PD, and a decrease in serum Cu 1 year after PD to be associated with an increased risk of developing NAFLD (Table 4).

**Table 4:** Risk factors for NAFLD 1 month, 6 months and 1 year after PD by multivariate analysis

	SE	Odds ratio	95% CI	*P* value
1 mo				
Age (not elderly)	.047	.911	.831‐.998	.002
Sex (female)	.892	11.496	2.002‐66.013	.031
Copper (1 mo)	.024	.948	.904‐.995	.002
6 mo				
Sex (female)	.586	5.899	1.869‐18.618	.002
Copper (6 mo)	.015	.962	.933‐.991	.011
1 y				
Copper (1 y)	.018	.942	.910‐.976	.001

CI, confidence interval; NAFLD, nonalcoholic fatty liver disease; PD, pancreatoduodenectomy.

## 4 DISCUSSION

Individual prevalence of NAFLD at 1 month, 6 months and 1 year after PD was 12%, 21% and 15%. These values seemed to be lower than those reported in previous studies^2^, ^3^, ^4^, ^5^, ^6^, ^11^, ^12^ because the development of NAFLD was not evaluated at a fixed time in most of the previous studies. Indeed, we recognized that several patients recovered from NAFLD spontaneously and others deteriorated sequentially, even though they were all taking the same pancreatic enzyme supplements for 1 year (Figure 2). Which patients were likely to suffer or recover from NAFLD remains uncertain from our study, but this is a first report documenting the sequential change in NAFLD development after PD.

PD can cause a diverse group of diseases as a result of impaired gut motility, insufficiency of neuroendocrine hormones, or malabsorption of certain trace elements. Previous reports found pancreatic head cancer to be an independent risk factor for the development of post‐PD NAFLD,^2^, ^3^ which was in accord with our results from 6 months after PD. Massive resection of the proximal pancreatic parenchyma is often necessary to obtain surgical curability of PDC, which results in insufficient exocrine function of the remnant pancreas. Nakagawa et al.^6^ reported that postoperative pancreatic exocrine insufficiency measured by 13C‐labeled mixed triglyceride breath test was closely associated with NAFLD. Pancrelipase delayed‐release supplement (LipaCreon^®^ 1200 mg/day) could improve exocrine dysfunction and was reported to have a significant beneficial effect on NAFLD after PD.^7^, ^13^ However, the prevalence of NAFLD has not decreased since 2012 when we started giving pancrelipase supplements in our hospital. A certain percentage of patients suffered NAFLD even if they received pancrelipase supplements, so we believe that the advantage of this drug is equivocal for the prevention of NAFLD development although it is helpful for intestinal digestion and absorption.

The most notable finding of the present study was that female sex was one of the most serious risk factors for the development of post‐PD NAFLD. The number of women was significantly higher in the NAFLD group than in the control group at all three time points after PD by univariate analysis. Multivariate analysis also showed that female sex was one of the risk factors at 1 month and 6 months after PD, so this appears to be quite an important factor associated with the development of post‐PD NAFLD. Song et al.^5^ reported that female sex was a significant risk factor but did not describe the reason for or emphasize this important finding in their report. Sato et al.^12^ also showed evidence that female sex was one of the risk factors for the development of post‐PD NAFLD by multivariate analysis as did the present study. They suggested the same mechanism in women as that of conventional NAFLD, which might be influenced by an imbalance in sex hormones after menopause in women over the age of 50.^16^ We also presume that the change in body fat distribution after menopause^17^ and the accumulation of visceral fat markedly accelerated by menopause^18^ can contribute to the high incidence of post‐PD NAFLD seen in women. Not elderly age, which was one of the risk factors for NAFLD at 1 month and 1 year after PD by univariate analysis in this series, might also be associated with the timing of menopause.

Cu is a trace element that is essential to the function of several enzymes involved in a variety of biological processes. Cu deficiency is known to affect the cardiovascular, musculoskeletal and nervous systems, to impair the metabolism of cholesterol and glucose and to affect the function of the hepatic antioxidative system.^19^ Aigner et al.^20^ reported that serum Cu levels were lower in patients with conventional NAFLD than in controls. Ackerman et al.^21^ suggested that Cu deficiency in rats was a link between a fructose‐enriched diet and the development of NAFLD. They also reported that decreases in plasma and hepatic Cu concentrations correlated with a decrease in the hepatic expression of copper‐zinc‐superoxide dismutase, disappearance of hepatic metallothionein and an increase in the expression of hepatic nitrotyrosine. Moreover, they showed the effects of antihypertensive and triglyceride‐lowering drugs on hepatic Cu concentrations in rats with NAFLD. Song et al.^22^ reported that liver fat accumulation was significantly induced in marginally dietary Cu‐deficient rats exposed to fructose. They suggested that high fructose‐induced NAFLD might be due, in part, to inadequate dietary Cu and that impairment of duodenum Cu transporter‐1 expression seen in fructose feeding might lead to decreased Cu absorption and subsequent Cu deficiency. In our study, a decrease in serum Cu was one of the most serious risk factors for NAFLD development at all three time points after PD by multivariate analysis. Therefore, we speculate that Cu supplementation might have therapeutic potential for post‐PD NAFLD ascribed to Cu deficiency.

Zn deficiency is one of the most consistent biochemical manifestations in alcoholic liver steatosis. Kang et al.^23^ reported that Zn supplementation reversed alcohol‐induced steatosis in mice, the mechanisms of which were by potentially inhibiting oxidative stress and reactivation of hepatocyte nuclear factor‐4α and peroxisome proliferators activated receptor‐α. However, from our results, Zn metabolism seemed not to contribute to the development of post‐PD NAFLD.

Although there is overwhelming evidence of an association between insulin resistance and the prevalence of conventional NAFLD,^24^ the value of HOMA‐R was not significantly different between the two groups and the prevalence of accompanying DM was paradoxically less in the NAFLD group than in the controls at 1 month after PD in this series. This is possibly because the probability of progressive stages of NAFLD, such as non‐alcoholic steatohepatitis (NASH) and liver fibrosis increases with the presence of impaired glucose tolerance.^25^ Hamaguchi et al.^26^ advocated that tight glycemic control might prevent histological progression, such as liver fibrosis in Japanese patients with NAFLD. Insulin resistance is probably not related to the development of post‐PD NAFLD itself but to the progression from NAFLD to NASH or more advanced stages.

Our results showed that a decrease in serum TP or TC level was one of the risk factors for NAFLD at any of the three time points after PD by univariate analysis. This factor indicates malnutrition of the patients, but whether this is a cause of NAFLD or a result of it was unclear. In this series, SSPPD was a significant risk factor from 6 months after surgery by univariate analysis. Nanashima et al.^27^ reported that the incidence of NAFLD was significantly higher in the patients who received SSPPD than in those receiving PPPD. In the late period, SSPPD could worsen the nutritional state of the patients more than PPPD.^28^ Elucidation of the molecular mechanisms may lead to an understanding of paradoxical hepatic fat accumulation in the malnourished state.

Little information about the influence of PVR on the development of post‐PD NAFLD was obtained. In this series, PVR imparted an increased risk of NAFLD from 6 months after PD by univariate analysis. This factor might worsen the portal venous flow in the late period after PD, which we did not evaluate in the present study.

In conclusion, NAFLD after PD frequently developed in women with a decrease in serum Cu and was influenced by various factors related to malnutrition involving pancreatic exocrine insufficiency such as PDC, SSPPD, PVR and high frequency of defecation. Because of the limitations of small sample size and the single‐institution nature of this study, further prospective or multi‐institutional joint research will be necessary to clarify the detailed mechanism of post‐PD NAFLD.

## DISCLOSURE

No grants, equipment, drugs, or other support was received that facilitated conduct of the work described in this article or the writing of the article itself.
